# Squamozygomatic mastoiditis

**DOI:** 10.1016/S1808-8694(15)30613-3

**Published:** 2015-10-18

**Authors:** Patrícia de Pinho Marques Araújo, Janini Oliveira Matos, Felipe Barbosa Madeira, Anderson de S. Araujo, Andréia Migueres Arruda, Shiro Tomita

**Affiliations:** 1 Third year otorhinolaryngology resident from the Federal University of Rio de Janeiro - UFRJ.; 2 Second year otorhinolaryngology resident - UFRJ.; 3 Third year otorhinolaryngology resident - UFRJ.; 4 MD. General practitioner - UFRJ.; 5 Third year otorhinolaryngology resident - UFRJ.; 6 Full Professor of Otorhinolaryngology - Federal University of Rio de Janeiro.

**Keywords:** complications of acutes otitis media, mastoiditis, squamozygomatic mastoiditis

## Abstract

Acute atypical mastoiditis, with temporal and/or facial edema, is called squamozygomatic mastoiditis. There are only a few reports of this occurrence in the literature, which occurs because of an inflammatory process spread to the zygommatic apophysis, when mastoid pneumatization reaches the zygoma or the squamous portion of the temporal bone. Diagnosis is made based on clinical history, physical exam and mastoid CT scan. Treatment is carried out with antibiotic therapy and surgery.

**Aim:**

to present a case of squamozygomatic mastoiditis and review the literature. Patients and methods: report of a case treated in our hospital during the year of 2003 and literature review through the Internet, we also reviewed otolaryngology books from known authors.

**Discussion:**

squamozygomatic mastoiditis is an atypical mastoiditis in which the inflammatory process spreads to the zygomatic apophysis. The infection reaches the temporal bone squamous portion and makes a fistula between this portion and the temporal muscle, shifting the pinna of the ear downwards and it may reach the face, eyes and eyelids. Diagnosis is carried out by clinical history, physical examination and mastoid CT Scan. Treatment is surgical, associated with antibiotic therapy.

## INTRODUCTION

Acute mastoiditis is an acute otitis media complication and currently has a lower incidence when compared to the pre-antibiotic era; consequently the atypical mastoiditis presentations have become proportionally rarer.

An acute mastoiditis may occasionally present itself as a temporal and/or facial edema. In these cases, the mastoid air cells inflammation may spread through the cells from the zygoma arch root cells toward adjacent soft tissue, such as, for example, the temporal muscle, being called squamous-zygomatic mastoiditis.

The goal of the present paper is to describe a case of squamous-zygomatic mastoiditis and to review the literature.

## OBJECTIVE

In this paper, the authors aim at presenting a case of squamous-zygomatic mastoiditis and to review the literature.

## PATIENTS AND METHODS

Report on a case evaluated in our hospital in 2003 and literature review through the Internet using the key words: mastoiditis, temporal osteomyelitis, and we also used otorhinolaryngology books from renowned authors.

## CASE REPORT

L.R.C., female, 50 years, Caucasian, with chronic renal insufficiency, under hemodialysis, evaluated by our Otorhinolaryngology Department in December of 2003 with progressive right temporal hypoacusis, otalgia and otorrhea on the right side, low fever (38°C), edema and pain, with symptoms that had started two weeks before. She did not complain of visual changes. During her examination, we found a bulging in her right temporal region with supra-auricular fluctuations and pinna shifting downwards. Her otoscopic exam was normal on the left side and she had an important edema in her external auditory meatus, preventing the introduction of an ear speculum, and thick otorrhea in small amounts. Her neurological exams were also normal.

Her mastoid and cranium contrasted CT scan showed an increase in cystic soft tissue in her right side temporal region with peripheral contrast uptake, and right-side mastoid cells obliteration. Small right temporal lytic lesion, near the mastoid, with contrast uptake at the adjacent dura mater.

Lab exams showed 18% hematocrit, 6g/dl hemoglobin, 4600 leucocytes/mm^3^ (0% basophiles, 1% eosinophils, 0% myelocytes, 0% metamyelocytes, 4% rods, 82% segmented, 11% lymphocytes, 2% monocytes), VHS 60 mm/h, urea 74mg/dl, creatinine 5.4 mg/dl, glucose 80 mg/dl.

The temporal mass was punched and we had a green purulent secretion oozing out - about 30 ml - which was sent for microbiological exam.

We started her on broad spectrum antibiotic therapy with 500mg Imipenem bid (corrected dose for patients with renal insufficiency under hemodialysis).

In two days, the patient evolved with pain worsening and edema increase in the temporal region, extending to the periorbital region; then emergency surgery was indicated.

**Figure 1:**
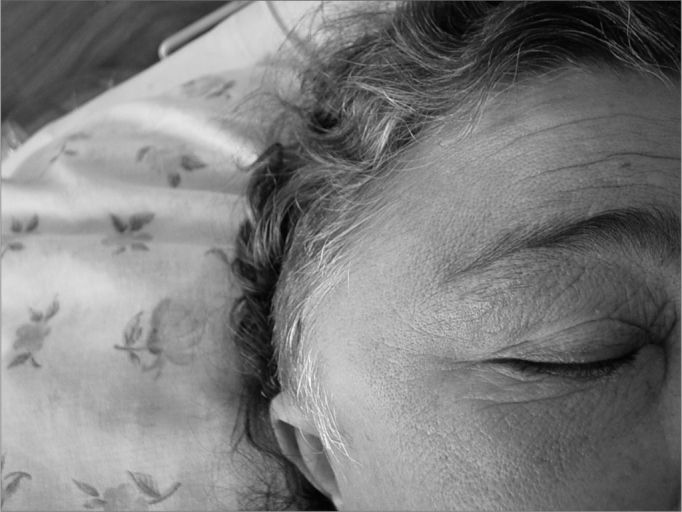
Bulging in the right temporal region with signs of fluctuation and pinna shift downwards. Facial edema and hyperemia.

**Figure 2:**
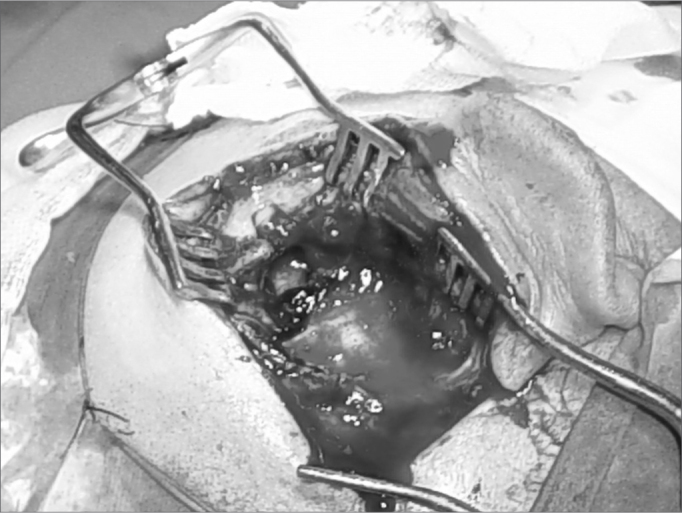
Surgical image – retroauricular approach, we found a thick greenish secretion, a large area of tissue necrosis, bone erosion with exposure of the dura (which was intact).

**Figure 3:**
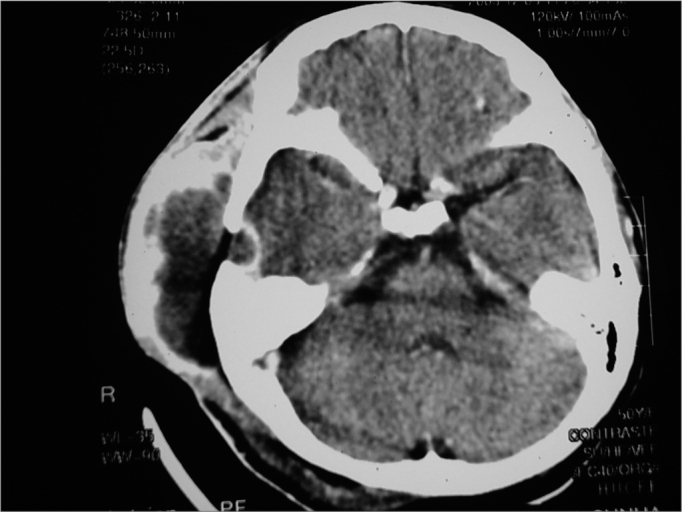
Mastoid CT scan, with a cystic soft tissue mass on the right temporal region, with peripheral contrast uptake, temporal lytic lesion on the right side with contrast uptake by the underlying dura matter.

The patient was operated upon, through the retroauricular approach, and we found a thick and greenish secretion, a large area of tissue necrosis, bone erosion and dura-mater exposure, which, by the way was intact. We cleaned and drained the area. We sent the material to microbiology and histopathology.

Hemoculture result: Streptococcus pneumoniae, sensitive to Ampicillin. After six days of Imipenem, and following the result of the hemoculture, the antibiotic was exchanged for 1g Ampicillin bid (corrected dose for renal insufficiency in a hemodialysis program).

Scintigraphy with technetium showed an increase in abnormal uptake in the right temporal lobe, and the diagnosis was of temporal osteomyelitis.

Pathology exam of the surgical specimen did not show neoplasia.

Patient had pain release, facial and temporal edema reduction and was discharged after three weeks of hospital stay, and was followed in an outpatient basis with oral antibiotics - 500mg amoxicillin PO, once a day (corrected dose for renal insufficiency in a hemodialysis program), for twelve weeks.

She kept on weekly outpatient follow up during the first month after hospital discharge, with a clear progressive improvement. During the next three months, she was followed up monthly, without complaints. A control scintigraphy with technetium and gallium was done in May of 2004, when there was a mild contrast build up in the right temporal region. A low probability test for osteomyelitis.

The patient is currently under outpatient follow up.

## DISCUSSION

The classic manifestation of acute mastoiditis is based on pain, hyperemia and retroauricular edema, with pinna protrusion, usually after an episode of upper airway infection. Symptoms of otalgia, headache, rhinorrhea and hearing loss are common. Low fever is usually present. In otoscopy there usually is evidence of acute otitis media (1/2).

Squamous-zygomatic mastoiditis is an atypical form of mastoiditis and is more frequent in children. It is the propagation of the inflammatory process down to the zygomatic apophysis root when the mastoid pneumatization reaches the zygoma and/or the squamous portion of the temporal bone. The infection hits the temporal bone squamous process and ends up creating a fistula between the latter and the temporal muscle (1/4). The purulent collection is deep in the temporal area and may follow the internal face of the temporal muscle and exteriorize in the masseter or genian zygomatic regions. After organized, the abscess pushes the pinna downwards and the collection reaches the face, eyes and eyelids. The patient complains of a painful pressure in the temporal region and there may be trismus (1/3).

Diagnosis is made following a detailed history and physical exam, radiological images with contrasted and not contrasted CT scan of the mastoid bones (1).

Culture material must be obtained as fast as possible in order to better guide the use of antibiotics (1). Material for culture can be obtained through tympanocentesis or an aspirate of the supra-auricular region in case of fluctuation. Hemoculture can be ordered, however it is rare to be able to isolate the major agent involved, Streptococcus pneumoniae.

The flora responsible for acute mastoiditis in both the classic and atypical manifestations of the disease is similar, however not identical as far as the bacteria that cause acute otitis media is concerned. The most frequently found pathogen is the Streptococcus pneumoniae, followed by Streptococcus pyogenes, Staphylococcus aureus and Haemophilus influenzae. Anaerobes such as bacteroid and Peptostreptococcus, gram-negative bacilli such as Pseudomonas, Klebsiella, Escherichia coli and Proteus are also less frequent agents (5).

The surgical treatment is indicated in the atypical manifestations of mastoiditis and must be associated with empirical antibiotic-therapy with a proper antibiotic agent against the bacteria found in the acute mastoiditis, until the results from the microbiology exams point to the most efficient antibiotic to be used against the right bacteria (1).

Squamous-zygomatic mastoiditis and other atypical manifestations, because they are rare, may be diagnosed later, causing harm to the patient such as a possible temporal osteomyelitis (4).

Temporal osteomyelitis is a rare entity in clinical practice. It is, in general, a complication of infectious processes that involve the temporal bones, such as acute and chronic otomastoiditis, cholesteatomatous chronic otitis media, and malignant otitis media. Although it may evolve fatally, causing fast death by intracranial complications, temporal osteomyelitis, seen during cases of otitis media and acute mastoiditis, almost always evolve more silently, giving some time to deter the infectious process by means of surgery^1^, ^3^. CT scan and MRI are considered the best option to define the anatomical extension of the disease. Scintigraphy with technetium is an excellent indicator of bone function. It shows high radio-isotope uptake in regions of intense osteoblastic activity, secondary to inflammatory or neoplastic processes. They remain positive for long periods of time, and this makes it inappropriate for cure control. Gallium scintigraphy helps identify whether the osteomyelitis process is active or inactive, since it is deposited in areas of active infection. It is an excellent test used to monitor treatment response and to decide when to interrupt it. Some authors advocate antibiotic use until gallium uptake returns to normal^1^, ^6^. Surgical treatment aims at removing all the bone tissue under suspicion of being affected by osteomielitis^4^.

## FINAL REMARKS

Today, acute otitis media bears a lower incidence of intra and extra cranial complications when compared to the pre-antibiotic era. Acute mastoiditis in its classical and atypical manifestations became proportionally rarer, especially the atypical manifestations, such as squamous-zygomatic mastoiditis, which was frequently discussed in the literature of the 40's; however, today there are very few reports of this clinical manifestation.

Otorhinolaryngologists must know and remember the possibility of having atypical mastoiditis, such as the squamous-zygomatic that may happen with temporal and facial edema, temporal region pain and trismus.
